# Associations of Probiotic Fermented Milk (PFM) and Yogurt Consumption with *Bifidobacterium* and *Lactobacillus* Components of the Gut Microbiota in Healthy Adults

**DOI:** 10.3390/nu11030651

**Published:** 2019-03-18

**Authors:** Noemí Redondo-Useros, Alina Gheorghe, Ligia E. Díaz-Prieto, Brenda Villavisencio, Ascensión Marcos, Esther Nova

**Affiliations:** Immunonutrition Group, Department of Metabolism and Nutrition, Institute of Food Science, Technology and Nutrition (ICTAN), Spanish National Research Council (CSIC), Jose Antonio Novais, St.10., 28040 Madrid, Spain; noemi_redondo@ictan.csic.es (N.R.-U.); draguta1@hotmail.com (A.G.); ldiaz@ictan.csic.es (L.E.D.-P.); brendavi@ucm.es (B.V.); amarcos@ictan.csic.es (A.M.)

**Keywords:** probiotic fermented milk, yogurt, healthy adults, gut microbiota, Bifidobacteria

## Abstract

The current study investigates whether probiotic fermented milk (PFM) and yogurt consumption (YC) are related to both the ingested bacteria taxa and the overall gut microbiota (GM) composition in healthy adults. PFM and YC habits were analyzed in 260 subjects (51% male) by specific questionnaires, and the following groups were considered: (1) PFM groups: nonconsumers (PFM-NC, n = 175) and consumers (PFM, n = 85), divided as follows: *Bifidobacterium*-containing PFM (Bif-PFM; n = 33), *Lactobacillus*-containing PFM (Lb-PFM; n = 14), and mixed *Bifidobacterium* and *Lactobacillus*-containing PFM (Mixed-PFM; n = 38); (2) PFM-NC were classified as: yogurt nonconsumers (Y-NC; n = 40) and yogurt consumers (n = 135). GM was analyzed through 16S rRNA sequencing. PFM consumers showed higher Bifidobacteria taxa levels compared to NC, from phylum through to species. Specifically, Bif-PFM consumption was related to higher *B. animalis* levels (*p* < 0.001), whereas Lb-PFM consumption was associated to higher levels of *Bifidobacterium* (*p* < 0.045) and *B. longum* (*p* = 0.011). YC was related to higher levels of the yogurt starter *Streptococcus thermophilus* (*p* < 0.001). Lactobacilli and the overall GM were not related either to YC or PFM consumption. According to these results, healthy adults might benefit from PFM intake by increasing *Bifidobacterium* levels.

## 1. Introduction

The definition of what is a healthy gut microbiota composition has been the subject of intense debate during recent years. On one side, current evidence has supported that healthy individuals share a common core of gut microbial genes, mainly belonging to the Firmicutes and Bacteroidetes phyla [1]. On the other side, a high variability in the gut microbiota composition among healthy individuals has been reported [2], revealing that in addition to prevalent species, each individual harbors a particular and variable number of bacterial species which are rare among individuals, defined as the “variable” gut microbiota [3]. Some of them appear to be autochthonous species that stably colonize the intestine, while others are allochthonous species, which have shown the ability to transiently integrate into the resident gut microbiota communities [4]. These transient bacteria are ingested as dietary ingredients, with yogurts and probiotic fermented milk (PFM) products being some of the main sources [5]. *Streptococcus thermophilus* and *Lactobacillus delbrueckii* subsp. *bulgaricus* from yogurt and other lactic acid bacteria (LAB) used in milk souring and Bifidobacteria strains from PFM products are mainly involved [6]. In this regard, gut microbiota analyses on ileum, colon mucosa, and fecal samples have revealed that some of the *Lactobacillus* species most commonly added to fermented dairy products, such as *Lb. plantarum*, *Lb. rhamnosus*, *Lb. paracasei*, and *Lb. casei*, appear to be transient microbes [4], as well as *Bifidobacterium animalis*, whose presence in feces increases during the PFM intake period, but decreases after one week of PFM cessation [7].

Despite their “transient” feature, ingested bacteria from dietary sources are believed to impact the composition and activity of the commensal gut communities, which is a known target for probiotic strains [6]. However, this assumption must not be generalized to all ingested species, since it mainly depends on bacterial species’ survival through the gastrointestinal tract and their persistence at high amounts in the intestine [8]. In this sense, while several studies have failed to show a good recovery of the yogurt starters in feces of healthy adults [9,10,11], others have shown the opposite [12,13]; differences which might be explained by the type of strain, the dose of ingested cells, or the different methodologies employed to assess the gut microbiota composition [14]. Despite these controversies, the most common effect observed after yogurt intake in healthy adults is an unaltered gut microbiota [9,15,16]; only one study has evidenced increased *Lactobacillus* levels [11], so the use of the term “probiotic” for yogurt starters based on their ability to beneficially affect the gut microbiota composition remains unclear. In this sense, the addition of probiotic strains along with yogurt bacteria during fermentation could facilitate a better colonization of the host intestinal environment [17]. Probiotic-based studies in healthy adults have shown a wide variety of findings, ranging from a lack of any significant change in the overall gut microbiota structure [18,19,20] to increases in the taxa level of the fed bacteria, mainly *Lactobacillus* and *Bifidobacterium* genera or species levels [21,22,23], or even changes in other bacterial groups [23,24]. The modulation of beneficial gut bacteria, whether by increases in *Lactobacillus*, *Bifidobacterium*, or other bacterial groups, seems to be desirable in order to achieve a good balance of the gut microbial communities at the expense of more harmful bacteria [25]. Consequently, beneficial effects in some gastrointestinal functions related to the stability of gut microbiota can be observed, such as the regulation of the intestinal transit time, which in turn might give improvements in the occurrence of constipation and intestinal symptoms [22,26], as well as in the gut immune system, protecting the host from infections, inflammatory processes, and other immune-related disorders [27]. Thus, given the importance of these effects for health maintenance and disease prevention in healthy populations [28], coupled with the fact that commensal microbes are highly sensitive to environmental factors such as dietary changes [29], the consumption of yogurt and PFM products might be a key strategy by which to achieve a beneficial gut microbiota composition, which is even more important nowadays, as lifestyle habits (e.g., diet, physical activity) have progressively become more unhealthy worldwide [30]. Therefore, we hypothesized that the consumption of PFM and yogurt is related to the taxa level of ingested bacteria in the gut microbiota of healthy adults. Thus, specifically, the main objective of this study is to evaluate the associations of PFM consumption with *Lactobacillus* and *Bifidobacterium* levels, as well as yogurt consumption with *Lactobacillus* and *Streptococcus* levels in healthy adults. As secondary objectives, the relationship between PFM and yogurt consumption with the overall gut microbiota composition was also investigated.

## 2. Materials and Methods

### 2.1. Subjects

A total of 272 adults aged between 25 and 50 years and with body mass index (BMI) between 18.5 and 35 kg/m^2^ were recruited for the study through advertisements in the university area and from several enterprises which disseminated the study among their employees as health-promoting campaigns. Exclusion criteria were: (1) suffering any of the following chronic diseases: type 1 diabetes; cancer; hepatic, cardiac, renal, pulmonary, or neurological diseases; congenital metabolic diseases; autoimmune diseases (including thyroid diseases); inflammatory bowel diseases; human immunodeficiency virus (HIV) and Cushing’s syndrome; or diagnosed food intolerances; (2) prescription of chronic medication; (3) antibiotic use in the last two months; (4) following a dietary regimen; (5) having undergone a surgical procedure in the last month.

Sample size was calculated taking Bifidobacteria as the outcome variable, with a difference of ±10% in Bifidobacteria levels being counted as significant. For this purpose, considering a mean of 9.8 log colony-forming units (cfu)/g dry weight feces and a standard deviation (SD) of 0.4 according to [31], for an alpha error of 0.05 and 80% power, 262 volunteers were necessary. From the total 272 recruited volunteers, 12 subjects dropped out from the study due to personal issues, resulting in a final sample size of 260 subjects (134 men and 126 women). Informed consent was obtained from each subject, and ethical approval was obtained from the Ethics Committee from Puerta de Hierro University Hospital (Madrid, Spain), together with the CSIC Bioethics Committee. The study was carried out according to the Declaration of Helsinki (64ª Asamblea General, Fortaleza, Brasil, October 2013) and Good Clinical Practices.

### 2.2. Experimental Design

The study design of this observational study involved two visits, during which trained nutritionists collected data about lifestyle habits in face-to-face interviews with the participants. In the first visit, overall health status, diagnosed diseases, drug administration, smoking habits, and sleep quality were evaluated through the Spanish National Health Survey questionnaire. Interviewer-assisted self-estimation of the total capital owned was used for the socioeconomic status (SES) classification as follows: (1) “low–intermediate”: income between 10,000 to 50,000 €/year; (2) “intermediate–high”: between 50,000 to 200,000 €/year; and (3) “high”: above 200,000 €/year. Height (Soehnle), waist circumference (Seca), body weight, and bioimpedance analysis (Tanita BC 601) without shoes and with light clothing were also measured in this first visit. Since the body mass index (BMI = weight (kg)/height (cm)^2^) does not represent an accurate measurement of body fat, optimal body fat percentage ranges were considered separately for men and women and subjects were divided into two groups: (1) normal weight (BMI <25 kg/m^2^) or overweight (BMI = 25–30 kg/m^2^) plus normal body fat percentages (21–32% for women and 10–20% for men); (2) overweight (BMI = 25–30 kg/m^2^) or obese (BMI = 30–35 kg/m^2^) plus high body fat percentages (>32% for women; >20% for men). Cutoff criteria for body fat percentages were taken from the Tanita bioimpedance equipment user manual.

**Figure 1:**
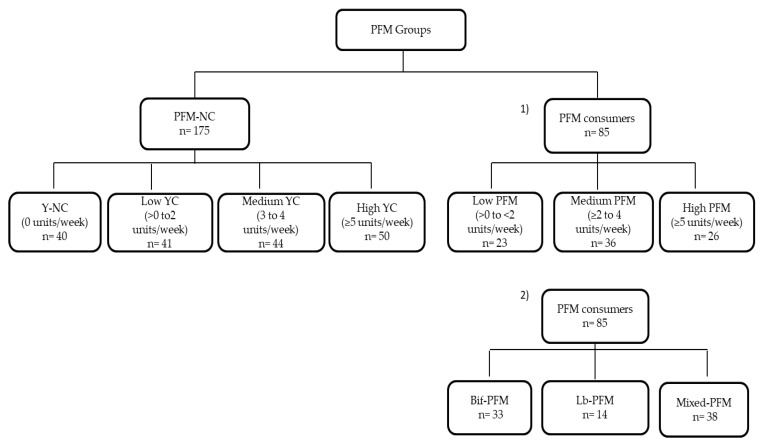
Subjects’ distribution into PFM groups. Y: yogurt; YC: yogurt consumption; PFM: probiotic fermented milk; NC: nonconsumers; Bif-PFM: Bifidobacterium-containing PFM; Lb-PFM: Lactobacillus-containing PFM; Mixed-PFM: mixed Bifidobacterium and Lactobacillus-containing PFM.

Subjects were instructed to collect a stool sample in sterile conditions and bring it frozen to the study center in a second visit, during which questionnaires about intestinal and dietary habits were also completed. In particular, intestinal symptoms were evaluated by a gastrointestinal symptom rating scale (GSRS) [32]. Subjects were asked about the frequency (0 = never, 1 = hardly ever, 2 = sometimes, and 3 = many times) and intensity (0 = none, 1 = light, 2 = moderate, and 3 = severe) of bloating, borborygmi, abdominal pain, burning, and flatulence. An individual score for each symptom and a total score of intestinal symptoms were subsequently calculated as the sum of both frequency and intensity scores. In addition, a validated food frequency questionnaire, which estimates the amounts and frequency of consumption of 104 items including both foods and beverages over the past one-year period, was completed with an interviewer-administration method [33], as well as an ad-hoc questionnaire on PFM and yogurt consumption. The type of products separately quantified included PFM containing *Bifidobacterium* species, PFM containing *Lactobacillus* species, and yogurt. The frequency of consumption was registered as never or almost never, 1 to 3 times per month, 1 to 6 times per week or (x) number of times per day. For group classification, PFM and yogurt consumption habits were analyzed on a weekly basis, considering the number of units reported separately for both PFM and yogurt groups. For PFM consumption, subjects were classified as nonconsumers (PFM-NC; n = 175) and PFM consumers (n = 85). Furthermore, two criteria were considered within PFM consumers: (1) Depending on the number of PFM products consumed, consumers were classified into three groups: (a) Low PFM (>0 to <2 PFM products/week; n = 23); (b) Medium PFM (≥2 to 4 PFM products/week; n = 36); (c) High PFM (≥5 PFM products/week; n = 26); (2) Depending on the type of PFM product, consumers were subclassified into three categories: (a) exclusive consumers of *Bifidobacterium*-containing PFM (Bif-PFM; n = 33); (b) exclusive consumers of *Lactobacillus*-containing PFM (Lb-PFM; n = 14); (c) consumers of both *Bifidobacterium* and *Lactobacillus*-containing PFM (Mixed-PFM; n = 38). Regarding PFM-NC, they were divided into 4 groups of yogurt consumption: Nonconsumers (Y-NC) (0 yogurts/week; n = 40), Low YC (>0 to 2 yogurts/week; n = 41), Medium YC (3 to 4 yogurts/week; n = 44), and High YC (≥5 yogurts/week; n = 50) (Figure 1).

### 2.3. Microbiota Analysis

The fecal samples were collected in sterile containers at home, immediately frozen at −20 °C, and transported on the next day in refrigerated conditions to the study center where they were stored at −80 °C until analysis. Bacterial DNA was extracted from 180–220 mg of each fecal sample using an optimized protocol [34], and DNA was finally recovered with the commercial kit QIAamp DNA Stool Mini Kit (Qiagen N.V., Venlo, The Netherlands) following the manufacturer’s instructions and frozen at −80 °C until further use. DNA concentration was measured by UV absorbance at 260 nm and the DNA quality was also assessed by the 260 nm/280 nm ratio using a Nanodrop ND-1000 spectrophotometer (Thermo Fisher Scientific, Wilmington, DE, USA). Subsequently, Picogreen analysis of double-stranded DNA was performed by a QuantiFluor ST fluorometer (Promega, Madison, WI, USA), and all samples were diluted to 0.5 ng/µL to be used in the amplification of the V3–V4 variable regions of the 16S RNA gene. DNA amplicon integrity was checked by 1.5% agarose gel electrophoresis (Pronadisa, Madrid, Spain), and libraries were normalized and pooled. Sequencing was performed by the MiSeq Illumina system (Illumina, San Diego, CA, USA) using the V3 kit on a 2 × 270 paired-end run. The taxonomy classification was performed with the MiSeq Reporter software (v2.3, Illumina) in several steps, including demultiplexing and FASTq file generation, obtaining a total of 37,793,518 pass-filter reads (144,803 mean reads/sample). Sequences were then clustered into operational taxonomic units (OTUs) with Classify Reads, a high-performance implementation of the Ribosomal Database Project (RDP) based on the Greengenes database, obtaining 20 phyla, 243 families, 651 genera, and 1492 species (Classify Reads accuracy was 100%, 99.97%, 99.65%, and 98.65%, respectively) [35]. Taxa with relative abundance <0.002% of total reads and also those with a prevalence of <10 subjects were removed, leaving a total of 11 phyla, 150 families, 241 genera, and 348 species for the statistical analysis. All sequence data were deposited in the Sequence Research Archive (SRA) under BioProject PRJNA523967.

### 2.4. Statistical Analysis

The Kolmogorow–Smirnov test was used to check the normality of the variables, and logarithmic transformation was applied for data normalization (variables: fiber (g/day), Actinobacteria, *Bifidobacteriaceae*, and *Bifidobacterium*). In this sense, variables fitting a normal distribution were expressed as mean ± standard deviation (SD), while variables not fitting a normal distribution were expressed as medians ± interquartile range (Q1–Q3). To assess differences in anthropometric and lifestyle characteristics between PFM groups, general linear models adjusted by BMI–fat groups and the covariables of age and energy when necessary were used for parametric variables, and the Mann–Whitney U test (MWU) for nonparametric variables and Chi-square test for categorical variables were also employed. Depending on the distribution of the data, the statistical analysis used to approach the primary hypothesis of the study included: (1) Generalized linear models to evaluate the relationship between PFM consumption and the phylum Actinobacteria and the *Bifidobacteriaceae* and *Bifidobacterium* groups using PFM groups, gender, and BMI–fat groups as fixed factors, and energy, total score of intestinal symptoms, and age as covariables; (2) Mann–Whitney U tests to assess the relationship between PFM consumption and *Bifidobacterium* species, *Lactobacillaceae* family, and *Lactobacillus* genus and species, and the Kruskal–Wallis (KW) test with Bonferroni correction to assess the relationship between YC and *Lactobacillaceae, Streptococaceae, Lactobacillus*, and *Streptococcus* genus and species. Previously, gender and BMI–fat effects were also assessed by MW U test and taken into account when necessary by splitting data before the analysis of the PFM effect. A false discovery rate (FDR) of 0.10 using the Benjamini–Hochberg procedure was used to control the false positive rate [36], considering as significant only those statistical tests whose *p* values were lower than their FDR critical value. The Chi-square test and z test were used for species-occurrence analysis of those *Bifidobacterium* species showing significantly different abundance between PFM groups. Spearman correlation was used to evaluate if the number of PFM products was related to Bifidobacteria taxa. In addition, the KW test with Bonferroni adjustment was conducted among the three levels of PFM consumption (low, medium, and high) for those variables showing a positive correlation with the number of PFM products consumed. On the other hand, the effect of the type of PFM product consumed was assessed by general linear models including the type of PFM (Bif-PFM, Lb-PFM, and Mixed-PFM), gender, and BMI–fat groups as fixed factors and age, energy, and total score of intestinal symptoms as covariables, with Bonferroni adjustment. *p* values < 0.05 were considered significant.

As the secondary hypothesis, the relationship of PFM and yogurt consumption with the overall gut microbiota composition (phyla, families, genus, and species), was assessed by MWU tests and the KW test with Bonferroni correction, respectively. Gender or BMI–fat groups were also taken into account whenever any of these factors showed a significant effect on a given taxa, considering a FDR of 0.10 as relevant. Data analysis was performed using SPSS v.23 Software (IBM Corp., Armonk, NY, USA).

## 3. Results

### 3.1. Subjects’ Distribution and Demographics, and Anthropometric and Lifestyle Characteristics of PFM Groups

The analysis of the subject distribution among PFM groups revealed that PFM consumers included both exclusive PFM consumers (n = 33) and mixed consumers (n = 52) who consumed both PFM and yogurts. This group included a similar proportion of subjects consuming more PFM units than subjects consuming more yogurt units (both, n = 20).

Regarding demographic characteristics, a higher proportion of women were PFM consumers compared to men (52/126 vs. 33/134, respectively; *p* = 0.004) (Table 1). The economic status as well as anthropometric and lifestyle characteristics were similar between male nonconsumers and male PFM consumers. Female PFM consumers did not differ in lifestyle and economic status compared to female nonconsumers, but showed lower BMI (*p* = 0.009), body fat (*p* = 0.001), and visceral fat (*p* = 0.032) and a trend to lower levels of waist circumference (*p* = 0.062) compared to female nonconsumers (Table 1).

### 3.2. Relationship of PFM Consumption with *Lactobacillus* and *Bifidobacterium* Levels

*Lactobacillaceae* and *Lactobacillus* genus and species levels were not different in PFM consumers compared to PFM-NC (data not shown). However, the phylum Actinobacteria (*p* = 0.011), the family *Bifidobacteriaceae* (*p* = 0.012), and the genus *Bifidobacterium* (*p* = 0.027) were higher in PFM consumers compared to PFM-NC (Table 2).

Despite 135 yogurt consumers being included in the PFM-NC group (Figure 1), no differences were found in Actinobacteria (*p* = 0.428), *Bifidobacteriaceae* (*p* = 0.392), and *Bifidobacterium* (*p* = 0.594) levels among YC groups (data not shown).

In this line, the higher *Bifidobacterium* levels found in PFM consumers could be attributed to the higher values observed in nine *Bifidobacterium* species, including *B. animalis*, *B. pseudolongum*, *B. thermophilum*, *B. merycicum*, *B. magnum*, *B. kashiwanohense*, *B. dentium*, *B. longum*, and *B. bombi* (Table 3). In addition, *B. animalis*, *B. pseudolongum*, *B. thermophilum*, *B. merycicum*, and *B. magnum* (the last one found only in men) were not only more abundant, but also showed higher ocurrence in PFM consumers, whereas the number of positive subjects for *B. kashiwanohense*, *B. dentium*, *B. longum*, and *B. bombi* did not differ between PFM-NC and PFM consumers.

**Figure 2:**
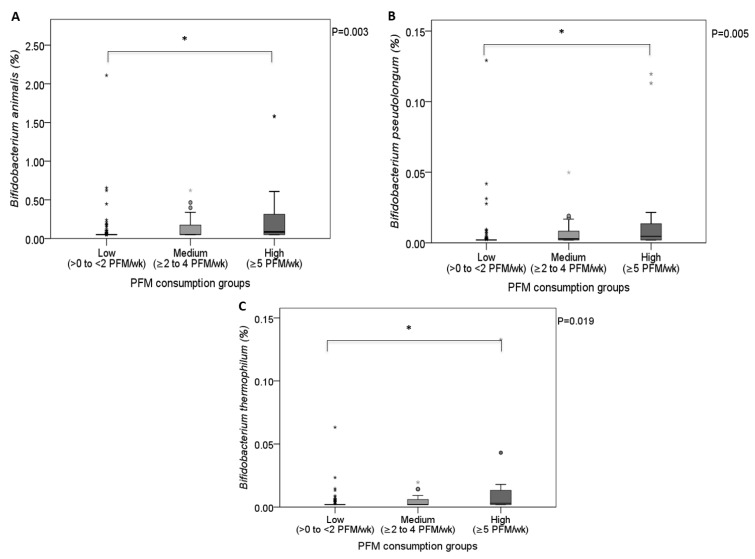
Relative abundance (%) of *B. animalis* (**A**), *B. pseudolongum* (**B**), and *B. thermophilum* (**C**) among PFM consumption groups. PFM consumption effect by KW test. * Pairwise comparisons with Bonferroni correction. Significance was set at *p* < 0.05.

At this point, we aimed to evaluate whether both the amount and the type of PFM consumed were driving the higher levels observed for Bifidobacteria taxa in PFM consumers. Thus, the number of PFM consumed was not significantly associated to the relative abundance of Actinobacteria (r = −0.002; *p* = 0.984), *Bifidobacteriaceae* (r = −0.003; *p* = 0.981), and *Bifidobacterium* (r = 0.005; *p* = 0.967), but did positively correlate to *B. animalis* (r = 0.405; *p* < 0.001), *B. pseudolongum* (r = 0.373; *p* < 0.001), *B. thermophilum* (r = 0.331; *p* = 0.002), *B. merycicum* (r = 0.247; *p* = 0.028), and *B. magnum* (men: r = 0.514; *p* = 0.002). As expected, the high consumption group showed greater levels of *B. animalis* (*p* = 0.002; Figure 2A), *B. pseudolongum* (*p* = 0.004; Figure 2B), and *B. thermophilum* (*p* = 0.015; Figure 2C) compared to the low-consumption group, but no differences were observed between the medium- and low-consumption groups. On the other hand, *B. magnum* and *B. merycicum* abundance did not differ among PFM groups of different consumption levels, despite the positive correlation found with the number of PFM products consumed. The different behavior observed in *Bifidobacterium* species in the three categories of PFM consumption might be dependent on the relative abundance found in the low-consumption group, since *B. animalis*, *B. pseudolongum*, and *B. thermophilum* were nearly absent in this group (Figure 2A–C), whereas abundance of *B. magnum* and *B. merycicum* in the low-consumption group was similar to those of the medium-consumption group (*B. magnum*, men: 0.001 (0.000–0.001) vs. 0.002 (0.002–0.007); *B. merycicum*: 0.002 (0.000–0.006) vs. 0.002 (0.000–0.021)).

**Figure 3:**
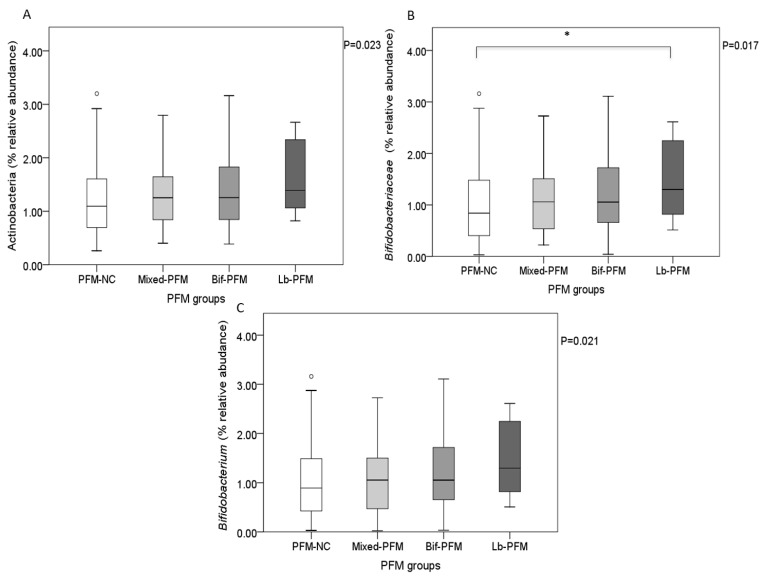
Relative abundance (%) of Actinobacteria (**A**), *Bifidobacteriaceae* (**B**), and *Bifidobacterium* (**C**) depending on the type of PFM product consumed. Effect of the type of PFM on Bifidobacteria taxa by generalized linear models. * Pairwise comparisons with Bonferroni correction. Significance was set at *p* < 0.05.

Regarding the type of PFM consumed, Lb-PFM consumers presented the higher levels found of *Bifidobacteriaceae* (*p* = 0.045 vs. PFM-NC; Figure 3B), with a trend towards higher levels of Actinobacteria and *Bifidobacterium* levels compared to PFM-NC (*p* = 0.069 and *p* = 0.056; Figure 3A,C, respectively).

This could be attributed to the higher levels found in Lb-PFM consumers of some of the most abundant species of *Bifidobacterium*, such as *B. longum* and *B. kashiwanohense*, compared to the rest of the groups (Supplementary Figure S1), reaching statistical significance compared to PFM-NC (*p* = 0.011 and *p* = 0.008, respectively; Table 4). In addition, the Mixed-PFM and Bif-PFM groups showed higher levels of *B. animalis*, *B. pseudolongum*, and *B. thermophilum* compared to PFM-NC and Lb-PFM consumers, and higher *B. merycicum* and *B. magnum* levels compared to PFM-NC. On the other hand, *B. bombi* and *B. dentium* abundance were not different among PFM groups (Table 4).

### 3.3. Relationship of YC with *Lactobacillus* and *Streptococcus* Levels

YC was not associated with *Lactobacillus* levels at any of the taxonomic levels analyzed (data not shown). However, *Streptococcaceae* levels tended to be higher in the high YC group compared to the low and Y-NC groups (median: 0.351 (0.152–0.623) vs. 0.161 (0.097–0.278) and 0.145 (0.074–0.452); *p* = 0.054 and *p* = 0.059, respectively). At the species level, significantly higher levels were observed in *S. thermophilus* levels between the high YC group and the low and Y-NC groups (median: 0.022 (0.007–0.065) vs. 0.0022 (0.001–0.008) and 0.000 (0.000–0.003) respectively; both *p* < 0.001), as well as in *S. fryi* and *S. milleri* levels between the high and the low YC groups (median: 0.011 (0.004–0.026) vs. 0.005 (0.002–0.013), *p* = 0.012; median: 0.030 (0.000–0.006) vs. 0.001 (0.000–0.005), *p* = 0.042, respectively).

### 3.4. Relationship of PFM and Yogurt Consumption with the Overall Gut Microbiota Composition

Regarding PFM consumption, PFM consumers showed lower levels of the Tenericutes phylum (*p* < 0.001; Supplementary Table S1) and the *Butyricimonas* genus (*p* < 0.001; Supplementary Table S2) compared to PFM-NC. There were no differences in families and species abundance between PFM groups (Supplementary Tables S3 and S4, respectively). In addition, YC was not related to any phyla, family, genus, or species analyzed (data not shown), except for those described in Section 3.3.

## 4. Discussion

The results of this study have shown that the relationship of probiotic fermented milk and yogurt consumption with the gut microbiota composition of healthy adults seems to be highly specific towards the bacterial genus and species that are present in the product, which is in agreement with a recent metanalysis of randomized clinical trials performed in healthy adults [37]. In this sense, Lactobacilli taxa did not show differences irrespective of consumption habits, whereas Bifidobacteria abundance was higher at all taxonomic levels analyzed in PFM consumers compared to nonconsumers. In this regard, a higher presence of several *Bifidobacterium* species was observed in PFM consumers. However, those species were among the lowest-abundance species out of all *Bifidobacterium* species found in the study subjects. Thus, *B. animalis*, *B. pseudolongum*, and *B. thermophilum*, which were nearly absent in low-amount PFM consumers, appear to have a dose-dependent relationship with the ingested probiotic strains. In this sense, a large number of studies have reported dose-dependent effects of probiotic bacteria on fecal recovery of the strains [38], whereas a less clear evidence exists in healthy adults about the effects of increased doses of probiotic bacteria on the modulation of gut microbial groups different from those administered [39,40]. A deeper study showed that the different probiotic strains present in the PFM products seem to be differently related to the Bifidobacteria taxa. In this line, the upper taxonomic levels, such as Actinobacteria, *Bifidobacteriaceae*, and *Bifidobacterium*, were more associated to the consumption of *Lactobacillus* containing PFM, which usually contain *L. casei* strains. Similar results regarding the *Bifidobacterium* genus have been found after the consumption of both *L. casei Zhang* and *L. casei shirota* over one month in healthy adults [22,24]. Furthermore, in the current study, the higher *Bifidobacterium* levels found in *Lactobacillus*-PFM consumers seem to be attributable to the greater levels of both *B. longum* and *B. kashiwanohense*, which represent the most abundant *Bifidobacterium* species out of those significantly related to PFM consumption. Since *B. longum* is the most abundant *Bifidobacterium* species in the intestinal niche of healthy adults [41], we suggest that *Lactobacillus*-containing PFM intake might modulate the autochthonous bacteria, which could represent a better health outcome than changes in allochthonous and transient bacteria [42]. Specifically studying the effect of *Lactobacillus*-containing PFM on gut *Bifidobacterium* species seems relevant according to the evidence mentioned above. The mechanisms involved might include bacterial crossfeeding through the breakdown of dietary carbohydrates [43] and the modulation of mucin polysaccharides synthesis and degradation [44,45] by *Lactobacillus* strains and their subsequent use by *Bifidobacterium* species. On the other hand, at the species level, the higher levels found of the low-abundance *B. animalis*, *B. pseudolongum, B. thermophilum, B. merycicum*, and *B. magnum* in PFM consumers seem to be due to the intake of *Bifidobacterium*-containing PFM, probably *B. animalis* strains, which are the most common species found in PFM products. Therefore, it seems that the intake of *B. animalis* strains might modulate phylogenetically close species almost absent in the study subjects and *B. animalis* levels itself, agreeing with other studies [9,18]. Despite *B. animalis* being considered a transient microorganism due to the low recovery in both fecal and mucosal samples [9,41], current evidence has pointed out its beneficial effects on the immune and gastrointestinal systems of healthy adults when administered in probiotic products [46,47]. However, the lack of evidence available about the presence and function of *B. pseudolongum*, *B. thermophilum*, *B. merycicum*, and *B. magnum* in humans [48], along with the nonzero probability of Illumina sequencing errors in base-calling as well as in reads alignment [49], which indeed might lead to OTU allocation errors between phylogenetically close species, impels us to be cautious about the association found between these species and the intake of PFM that contain *B. animalis*. Likewise, the consumption of yogurt was also associated to higher levels of the yogurt starter *S. thermophilus*, which has been related to probiotic properties in lactose intolerance conditions due to its β-galactosidase activity [50], as well as possibly to the reduction of gastrointestinal and allergy symptoms [51]. However, it should be kept in mind that these effects might be transient and return to baseline if the intake of probiotic and yogurt bacteria is discontinued [37], or even if the consumption is not frequent enough, as derived from our results showing that the consumption of more than five PFM products per week is associated with higher levels of certain *Bifidobacterium* species, while the consumption of more than 0 to 2 PFM products per week did not relate to higher levels of these species.

On the other hand, one of the main foci in probiotic-based studies is the evaluation of Lactobacilli changes, with evidence showing either positive [11,24,52] or neutral effects [15,16,18]. *Lactobacillus* species account for a very small proportion in the gut microbiota composition of our subjects, agreeing to previous works suggesting that Lactobacilli levels are very low in the colon and predominate in the small intestine [4,53]; thus, it is not surprising the lack of findings related to both probiotics containing fermented milks and yogurt intake in this respect. The limited outcomes observed in our study regarding the overall gut microbiota composition agrees to a recent systematic review of randomized clinical trials in healthy adults, which concluded that probiotic intake does not affect the α-diversity and richness of the gut microbiota of healthy adults [38], in accordance to other interventional studies showing no effects in the bacterial groups analyzed [7,18,54]. On the contrary, Ferrario et al. reported changes in the β-diversity of the gut microbiota after the consumption of *L. paracasei* DG compared to placebo [55], and studies by both Zhang et al. and Kim et al. revealed that probiotic consumption changed several bacterial groups distinct from the fed bacteria [19,24]. In our study, although the majority of the main bacterial groups were not related with the consumption of probiotic strains, some minor bacterial groups were lower in PFM consumers, such as Tenericutes and *Butyricimonas*, which have been previously related to adverse physiological situations, such as higher levels of the proatherogenic metabolite trimethylamine-*N*-oxide (TMAO) [56] and alcoholism [57], respectively. However, these assumptions must be made with caution due to the lack of evidence in healthy adults. One of the main factors possibly influencing the differences observed among studies might be the type of probiotic strain used, since specificity is a well-known feature of probiotics when referring to immune and intestinal health effects [58]. In addition, other factors should be taken into account when comparing data from different probiotic-based studies: (1) the methodology used to assess the 16S RNA gene, ranging from traditional molecular approaches to next-generation sequencing technologies providing a deeper analysis of gut microbial communities [59]; (2) the food matrix containing the probiotic strains [7,18,19]; (3) the diet composition of the host, and particularly the carbohydrate content, which represent one of the main energy sources for commensal bacteria [60]; (4) the gender of the study subjects [61,62] and their different dietary habits, as observed in the higher number of women consumers of PFM who showed a better anthropometric profile compared to women PFM-NC; and finally, (5) host intrinsic factors such as the basal gut microbiota composition [9], and several metabolic pathways affecting the host gut microbiota such as bile acid secretion [63] and the gut–enteric nervous system interaction mechanisms [64] might also influence the gut microbiota response to probiotics. Therefore, interventional studies performed separately in both healthy men and women investigating through metagenomics the effects of defined probiotic strains not only on gut microbiota changes, but also in the gut functionality and metabolic outcomes are warranted.

In conclusion, probiotic fermented milk and yogurt intake was associated with higher levels of the ingested bacteria. In particular, those pertaining to Bifidobacteria were positively associated with PFM intake and seem to be dependent on the type of probiotic strain present. However, further research is necessary to confirm whether *Lactobacillus*-containing PFM products promote the growth of specific *Bifidobacterium* species.

## Figures and Tables

**Table 1:** Demographic, anthropometric, and lifestyle characteristics in men and women stratified according to PFM groups.

	Nonconsumers	PFM Consumers			
**Men**	**n = 101**	**n = 33**	*p* *	***p*^#^**	***p*^†^**
Age (years)	37.31 (5.895)	35.84 (5.957)	–	–	–
Energy (kcal/day)	2204 (547.4)	2268 (619.6)	–	0.779	–
Carbohydrates (%)	40.3 (5.810)	39.53 (5.570)	0.371	–	–
Proteins (%)	17.44 (2.661)	17.89 (3.119)	0.370	–	–
Fat (%)	39.37 (5.068)	39.56 (4.26)	0.759	–	–
Fiber (g/day) ^Ʇ^	23.73 (10.15)	22.55 (6.520)	0.361	–	–
Sleep hours	7.285 (0.827)	7.355 (0.755)	–	0.820	–
BMI (kg/m^2^)	25.91 (3.201)	25.43 (3.570)	–	0.733	–
Body fat (%)	19.55 (5.616)	18.10 (5.914)	–	0.373	
Waist circumference (cm)	88.12 (8.838)	86.80 (8.451)	–	0.815	–
Visceral fat index	6.0 (4.0–9.0)	6.0 (4.0–7.5)	–	–	0.284
Total score of intestinal symptoms	7.0 (4.0–11.0)	9.0 (6.0–11.0)	–	–	0.345
Smoking habits (%)					
Current smokers	77.8	22.2	–	–	
Former smokers	83.3	16.7	–	–	0.634
Nonsmokers	73.0	27.0	–	–	
Income (%)					
Low (<50,000 euros/year)	34.7	30.3	–	–	
Medium (50,000–200,000 euros/year)	45.5	45.5	–	–	0.807
High (>200,000 euros/year)	19.8	24.2	–	–	
**Women**	**n = 74**	**n = 52**			
Age (years)	34.73 (6.623)	34.54 (6.592)	–	–	–
Energy (kcal/day)	2018 (470.9)	1992 (506.3)	–	0.721	–
Carbohydrates (%)	39.82 (7.853)	40.77 (6.750)	0.946	–	–
Proteins (%)	16.90 (2.941)	16.89 (2.618)	0.802	–	–
Fat (%)	41.01 (6.457)	40.26 (5.747)	0.913	–	–
Fiber (g/day) ^Ʇ^	23.03 (10.64)	22.58 (8.845)	0.623	–	–
Sleep hours	7.517 (0.841)	7.566 (0.624)	–	0.737	–
BMI (kg/m^2^)	23.41 (3.312)	22.02 (2.279)	–	**0.009**	–
Body fat (%)	28.85 (7.261)	25.04 (4.770)	–	**0.001**	–
Waist circumference (cm)	74.87 (8.442)	72.44 (5.475)	–	0.062	
Visceral fat index	3.0 (2.0–4.13)	2.5 (1.0–3.0)	–	–	**0.032**
Total score of intestinal symptoms	11.0 (7.0–15.0)	12.0 (6.0–16.0)	–	–	0.546
Smoking habits (%)					
Current smokers	66.7	33.3	–	–	
Former smokers	45.5	54.5	–	–	0.194
Nonsmokers	61.9	38.1	–	–	
Income (%)					
Low (<50,000 euros/year)	64.9	55.8	–	–	
Medium (50,000–200,000 euros/year)	32.4	32.7	–	–	0.120
High (>200,000 euros/year)	2.70	11.5	–	–	

* ^# †^ PFM consumption effect between PFM groups in both men and women by generalized linear models for parametric variables (* Model 1: adjusted by BMI–fat groups, age, and energy; ^#^ Model 2: adjusted by age), ^†^ Mann–Whitney U test for nonparametric variables and Chi-square test for categorical variables. Statistical significance was set at *p* < 0.05. Significant differences are marked in bold. ^Ʇ^ Variables were logarithmically transformed.

**Table 2:** Relative abundance (%, mean (SD)) of Actinobacteria, *Bifidobacteriaceae*, and *Bifidobacterium* between PFM groups.

	PFM Groups		
% Relative Abundance	Nonconsumers (n = 175)	Consumers (n = 85)	*p* Value *
Actinobacteria ^#^	2.945 (2.984)	4.089 (4.090)	0.011
*Bifidobacteriaceae* ^#^	2.368 (2.885)	3.358 (3.794)	0.012
*Bifidobacterium* ^#^	2.399 (2.869)	3.307 (3.773)	0.027

* PFM consumption effect by generalized linear models with PFM groups, gender, and BMI–fat groups as fixed factors and age, energy, and total score of intestinal symptoms as covariables. Significance was set at *p* < 0.05. ^#^ Variables were logarithmically transformed.

**Table 3:** Relative abundance (%, median (IQR)) and occurrence (number and % of positive subjects) of *Bifidobacterium* species showing significant differences between PFM groups.

PFM Groups							
	Nonconsumers (n = 175)		Consumers (n = 85)				
% Relative Abundance	Occurrence (n, %)	Median (IQR)	Occurrence (n, %)	Median (IQR)	*p* Value ^†^ Occurrence	*p* Value * Abundance	FDR Critical Value
*Bifidobacterium animalis*	41 (23.4)	0.000 (0.000–0.000)	49 (66.2)	0.011 (0.000–0.187)	<0.001	<0.001	0.027
*Bifidobacterium pseudolongum*	29 (16.6)	0.000 (0.000–0.000)	48 (64.2)	0.001 (0.000–0.007)	<0.001	<0.001	0.009
*Bifidobacterium thermophilum*	15 (8.6)	0.000 (0.000–0.000)	36 (50.7)	0.000 (0.000–0.007)	<0.001	<0.001	0.018
*Bifidobacterium merycicum*	102 (58.3)	0.001 (0.000–0.002)	66 (77.5)	0.005 (0.001–0.030)	0.002	<0.001	0.036
*Bifidobacterium kashiwanohense*	160 (91.4)	0.012 (0.002–0.043)	83 (97.6)	0.017 (0.005–0.061)	0.057	0.016	0.045
*Bifidobacterium dentium*	71 (40.6)	0.000 (0.000–0.001)	44 (52.1)	0.001 (0.000–0.001)	0.088	0.045	0.054
*Bifidobacterium longum*	170 (97.1)	0.175 (0.043–0.356)	79 (92.9)	0.176 (0.050–0.658)	0.114	0.050	0.063
*Bifidobacterium bombi*	170 (97.1)	0.009 (0.003–0.018)	84 (98.8)	0.012 (0.004–0.022)	0.397	0.059	0.072
*Bifidobacterium magnum* ^#^							
Men (n = 134: 101 NC/33 C)	55 (54.5)	0.001 (0.000–0.001)	26 (78.8)	0.001 (0.001–0.007)	0.013	<0.001	0.010
Women (n = 126: 74 NC/52 C)	49 (66.2)	0.001 (0.000–0.002)	42 (80.8)	0.002 (0.001–0.007)	0.073	<0.001	0.005

NC: Nonconsumers; C: Consumers. ^†^ Differences in species occurrence (number of positive and negative subjects) between PFM groups according to Chi-square test. Significance was set at *p* < 0.05. * PFM consumption effect according to Mann–Whitney U test. Those variables whose *p* values were lower than their false discovery rate (FDR) critical value were considered significant. ^#^ Gender significantly affected the taxa relative abundance.

**Table 4:** Relative abundance (%, median (IQR)) of *Bifidobacterium* species showing significant differences among PFM groups.

PFM Groups					
% Relative Abundance	PFM-NC n = 175	Bif-PFM n = 33	Lb-PFM n = 14	Mixed-PFM n = 38	*p* Value *
*Bifidobacterium animalis*	0.000 ^a^ (0.000–0.000)	0.018 ^b^ (0.001–0.241)	0.000 ^a^ (0.000–0.000)	0.002 ^b^ (0.000–0.157)	<0.001
*Bifidobacterium pseudolongum*	0.000 ^a^ (0.000–0.000)	0.001 ^b^ (0.000–0.012)	0.000 ^a^ (0.000–0.000)	0.001 ^b^ (0.000–0.006)	<0.001
*Bifidobacterium thermophilum*	0.000 ^a^ (0.000–0.000)	0.001 ^b^ (0.000–0.011)	0.000 ^a^ (0.000–0.000)	0.000 ^b^ (0.000–0.006)	<0.001
*Bifidobacterium merycicum*	0.001 ^a^ (0.000–0.002)	0.005 ^b^ (0.001–0.034)	0.001 ^ab^ (0.001–0.425)	0.004 ^b^ (0.001–0.028)	<0.001
*Bifidobacterium kashiwanohense*	0.012 ^a^ (0.003–0.043)	0.018 ^ab^ (0.006–0.049)	0.067 ^b^ (0.029–0.143)	0.016 ^ab^ (0.004–0.069)	0.009
*Bifidobacterium dentium*	0.000 (0.000–0.001)	0.001 (0.000–0.004)	0.000 (0.000–0.003)	0.001 (0.000–0.001)	0.184
*Bifidobacterium longum*	0.175 (0.043–0.357)	0.194 (0.042–0.982)	0.467 (0.141–1.300)	0.169 (0.069–0.515)	0.071
*Bifidobacterium bombi*	0.009 (0.003–0.018)	0.011 (0.005–0.027)	0.016 (0.008–0.036)	0.012 (0.004–0.018)	0.213
*Bifidobacterium magnum* ^#^					
Men	0.001 ^a^ (0.000–0.001)	0.002 ^b^ (0.001–0.011)	0.001 ^ab^ (0.000–0.001)	0.003 ^b^ (0.001–0.007)	<0.001
Women	0.001 ^a^ (0.000–0.002)	0.002 ^b^ (0.001–0.009)	0.001 ^ab^ (0.001–0.003)	0.004 ^b^ (0.000–0.009)	0.004

* PFM consumption effect by Kruskal–Wallis test of those species showing significant differences between PFM-NC and PFM consumers. Different superscripts mean that differences between PFM groups were found with post-hoc Bonferroni test (*p* < 0.05) ^#^ Gender significantly affected the taxa relative abundance.

## Supplementary Materials

The following are available online at https://www.mdpi.com/2072-6643/11/3/651/s1, Figure S1: Proportion of *Bifidobacterium* species showing significant differences among PFM groups depending on the type of product (scale relative to the relative abundance of the species shown); Table S1: Relative abundance [%; median (IQR)] of bacterial phyla between PFM groups; Table S2: Relative abundance [%; median (IQR) ] of the main bacterial genus (>0.5%), and those low-abundance genera (below 0.5%) showing significant differences between PFM groups; Table S3: Relative abundance [%; median (IQR)] of the main bacterial families (>0.5%) between PFM groups; Table S4: Relative abundance [%; median (IQR)] of the main bacterial species (>0.5%), and those low-abundance species (below 0.5%) showing significant differences between PFM groups.
