# Supplementary material for: Associations of Probiotic Fermented Milk (PFM) and Yogurt Consumption with Bifidobacterium and Lactobacillus Components of the Gut Microbiota in Healthy Adults
Source: Nutrients. 2019 Mar 18;11(3):651. doi: 10.3390/nu11030651 (PMC6470543; doi:10.3390/nu11030651)
Supplement: Supplementary file 1 [file nutrients-11-00651-s001.pdf]

Supplementary Materials

Associations of probiotic fermented milk (PFM) and classic yogurt consumption with *Bifidobacterium* and *Lactobacillus* components the gut microbiota in healthy adults

Table S1. Relative abundance (%; median (IQR)) of bacterial phyla in PFM groups.

	PFM groups			
% Relative abundance	Nonconsumers (175)	Consumers (85)	*p* value*	FDR critical value
Firmicutes[#]				
Men	60.21 (54.09–67.66)	63.87 (57.65-72.29)	0.089	0.075
Women	58.97 (53.12-64.46)	56.45 (48.59-61.69)	0.057	0.037
Bacteroidetes	24.16 (18.05-30.70)	23.32 (15.49-34.07)	0.787	0.085
Proteobacteria[#]				
Men	4.898 (3.903-5.750)	4.274 (3.132-5.489)	0.065	0.050
Women	5.332 (4.240-6.480)	4.782 (4.079-6.580)	0.269	0.100
Actinobacteria	1.987 (0.988-3.989)	2.575 (1.380-5.190)	0.017	0.028
Cyanobacteria	0.298 (0.195-0.498)	0.284 (0.168-0.671)	0.579	0.057
Verrucomicrobia	0.190 (0.011-0.938)	0.156 (0.008-1.213)	0.899	0.100
Tenericutes	0.115 (0.069-0.228)	0.075 (0.053-0.129)	**<0.001**	**0.014**
Synergistetes	0.077 (0.038-0.125)	0.0723 (0.038-0.097)	0.282	0.042
Spirochaetes[#]				
Men	0.006 (0.003-0.013)	0.009 (0.005-0.019)	0.045	0.025
Women	0.008 (0.005-0.017)	0.016 (0.006-0.020)	0.026	0.012
Acidobacteria	0.007 (0.001-0.006)	0.003 (0.001-0.006)	0.770	0.070
Fusobacteria[#]				
Men	0.002 (0.001-0.005)	0.002 (0.007-0.003)	0.073	0.062
Women	0.003 (0.001-0.005)	0.004 (0.002-0.008)	0.097	0.087

*PFM consumption effect according to Mann–Whitney U test. Those variables whose *p* values were lower than their FDR critical value were considered significant.

[#] Gender significantly affected the taxa relative abundance.

Table S2. Relative abundance (%; median (IQR)) of the main bacterial genera (>0.5%), and those low-abundance genera (below 0.5%) showing significant differences between PFM groups.

	PFM groups			
% Relative abundance	Nonconsumers (175)	Consumers (85)	*p* value*	FDR critical value
Bacteroides	12.67 (7.790-19.73)	13.75 (6.947-19.60)	0.894	0.089
Blautia	7.956 (6.199-9.767)	7.746 (6.085-9.618)	0.452	0.057
Faecalibacterium	7.408 (5.619-9.985)	8.169 (5.163-9.755)	0.756	0.079
Ruminococcus	4.624 (3.661-5.860)	4.516 (3.085-5.964)	0.438	0.050
Clostridium	4.114 (3.294-5.341)	3.974 (2.686-5.685)	0.274	0.036
Alkaliphilus#				
Men	1.901 (1.178-3.882)	2.070 (1.060-3.668)	0.953	0.094
Women	0.885 (1.950-3.430)	1.372 (0.738-2.474)	0.123	0.017
Roseburia#				
Men	2.027 (1.194-3.103)	2.005 (0.573-3.588)	0.782	0.081
Women	1.575 (0.860-2.957)	1.878 (0.714-2.861)	0.921	0.092
Lachnospira	1.877 (0.944-2.861)	1.820 (0.966-3.210)	0.709	0.081
Bifidobacterium	1.442 (0.509-3.488)	1.876 (0.887-4.506)	0.0	0.009
Coprococcus	1.892 (1.234-2.935)	1.623 (0.978-2.682)	0.191	0.031
Flavobacterium#				
Men	1.629 (0.792-2.706)	1.104 (0.527-1.847)	0.031	0.009
Women	2.126 (1.018-3.554)	2.046 (1.226-2.488)	0.375	0.047
Parabacteroides	1.267 (0.725-1.981)	1.141 (0.599-1.813)	0.155	0.027
Collinsella	1.019 (0.581-2.013)	1.068 (0.461-1.918)	0.560	0.062
Slackia	0.749 (0.416-1.381)	0.701 (0.303-1.475)	0.243	0.034
Sutterella	0.601 (0.271-1.188)	0.477 (0.217-0.944)	0.162	0.026
Others (<0.5%)				
Butyricimonas	0.233 (0.118-0.388)	0.142 (0.049-0.265)	**<0.001**	**<0.001**

*PFM consumption effect by Mann–Whitney U test. Those variables whose *p* values were lower than their FDR critical value were considered significant. # Gender significantly affected the taxa relative abundance.

Table S3. Relative abundance (%; median (IQR)) of the main bacterial families (>0.5%) in PFM groups.

	PFM groups			
% Relative abundance	Nonconsumers (175)	Consumers (85)	*p* value	FDR critical value
Lachnospiraceae	17.47 (14.80-21.61)	17.56 (6.082-17.13)	0.357	0.043
Ruminococcaceae	15.62 (13.41-18.61)	15.86 (12.71-19.40)	0.935	0.093
Bacteroidaceae	12.90 (7.809-19.73)	13.76 (6.947-19.60)	0.799	0.079
Clostridiaceae	9.665 (6.406-12.77)	8.486 (5.610-12.01)	0.100	0.020
Coriobacteriaceae	2.373 (1.548-4.051)	2.647 (1.167-4.101)	0.937	0.094
Veillonellaceae	1.991 (1.067-4.381)	2.057 (1.027-4.453)	0.766	0.076
Flavobacteriaceae[#]				
Men	1.677 (0.915-2.816)	1.307 (0.573 -1.972)	0.044	0.005
Women	2.281 (1.289-3.722)	2.229 (1.436-2.678)	0.472	0.055
Porphyromonadaceae	1.804 (1.191-2.669)	1.508 (0.836-2.496)	0.040	0.012
Bifidobacteriaceae	1.316 (0.487-3.397)	1.923 (0.918-4.529)	0.014	0.007
Erysipelotrichaceae	0.831 (0.442-1.466)	0.775 (0.448-1.643)	0.997	0.100
Alcaligenaceae	0.617 (0.276-1.191)	0.502 (0.243-0.993)	0.253	0.037
Sphingobacteriaceae[#]				
Normal body fat levels	0.719 (0.355-1.366)	0.617 (0.324-1.449)	0.573	0.070
High body fat levels	0.436 (0.250-0.760)	0.353 (0.223-1.637)	0.982	0.982

[#] Gender or BMI–fat groups significantly affected the taxa relative abundance.

Table S4. Relative abundance (%; median (IQR)) of the main bacterial species (>0.5%), and those low-abundance species (below 0.5%) showing significant differences between PFM groups.

	PFM groups			
% Relative abundance	Nonconsumers (175)	Consumers (85)	*p* value	FDR critical value
Blautia coccoides	1.991 (1.543-2.589)	1.856 (1.347-2.612)	0.315	0.038
Bacteroides vulgatus	1.488 (0.560-3.387)	1.269 (0.355-3.064)	0.305	0.036
Faecalibacterium prausnitzii	1.513 (0.924-2.160)	1.431 (0.611-2.172)	0.287	0.033
Alkaliphilus peptidifermentans	0.653 (0.228-1.309)	0.560 (0.240-1.692)	0.947	0.094
Bacteroides uniformis	0.877 (0.386-1.709)	0.822 (0.165-1.634)	0.302	0.034
Collinsella aerofaciens	0.760 (0.469-1.577)	0.836 (0.364-1.577)	0.624	0.065
Alkaliphilus crotonatoxidans	0.773 (0.301-1.730)	0.576 (0.241-1.079)	0.049	0.009
Lachnospira pectinoschiza[#]				
Normal body fat levels	1.227 (0.818-1.781)	0.939 (0.648-1.408)	0.021	0.012
High body fat levels	0.855 (0.533-1.238)	0.906 (0.432-1.615)	0.476	0.050
Roseburia faecis	0.751 (0.321-1.463)	0.758 (0.254-1.338)	0.624	0.065
Bacteroides rodentium	0.703 (0.343-1.356)	0.753 (0.191-1.325)	0.440	0.047
Ruminococcus bromii	0.657 (0.014-1.440)	0.120 (0.004-1.350)	0.113	0.016
Clostridium alkalicellulosi				
Men	0.724 (0.497-0.949)	0.774 (0.540-1.036)	0.655	0.070
Women	0.620 (0.470-0.970)	0.496 (0.383-0.663)	0.021	0.005
Oscillospiraeae	0.673 (0.396-0.918)	0.602 (0.403-0.818)	0.403	0.044
Bacteroides xylanisolvens	0.601 (0.330-0.983)	0.557 (0.230-0.953)	0.943	0.094
Parabacteroides distasonis	0.527 (0.204-0.955)	0.413 (0.154-0.840)	0.161	0.021
Others (<0.5%)				
Bifidobacterium thermophilum	0.000 (0.000-0.000)	0.000 (0.000-0.004)	**<0.001**	**0.001**
Bifidobacterium pseudolongum	0.000 (0.000-0.000)	0.001 (0.000-0.005)	**<0.001**	**0.001**
Bifidobacterium merycicum	0.001 (0.000-0.002)	0.003 (0.001-0.024)	**<0.001**	**0.001**
Bifidobacterium animalis	0.000 (0.000-0.000)	0.001 (0.000-0.147)	**<0.001**	**<0.001**
Bifidobacterium magnum[#]				
Men	0.001 (0.000-0.001)	0.002 (0.001-0.007)	**<0.001**	**0.002**
Women	0.001 (0.000-0.002)	0.002 (0.001-0.007)	**<0.001**	**0.001**

*PFM consumption effect by Mann–Whitney U test. Those variables whose *p* values were lower than their FDR critical value were considered significant.

[#] Gender or BMI–fat groups significantly affected the taxa relative abundance.
